# Symptomatic bone marrow lesions induced by reduced bone mineral density in middle-aged women: a cross-sectional Japanese population study

**DOI:** 10.1186/s13075-019-1900-4

**Published:** 2019-05-06

**Authors:** Seiya Ota, Daisuke Chiba, Eiji Sasaki, Gentaro Kumagai, Yuji Yamamoto, Shigeyuki Nakaji, Eiichi Tsuda, Yasuyuki Ishibashi

**Affiliations:** 1 Department of Orthopaedic Surgery, Hirosaki University Graduate School of Medicine, 5 Zaifu-cho, Hirosaki, Aomori 036-8562 Japan; 2 Department of Social Medicine, Hirosaki University Graduate School of Medicine, 5 Zaifu-cho, Hirosaki, Aomori 036-8562 Japan; 3 Department of Rehabilitation Medicine, Hirosaki University Graduate School of Medicine, 5 Zaifu-cho, Hirosaki, Aomori 036-8562 Japan

**Keywords:** Bone marrow lesion, Bone mineral density, Bone markers, Knee osteoarthritis

## Abstract

**Background:**

The etiology of bone marrow lesions (BMLs) without knee osteoarthritis (KOA) and their association with bone fragility are unclear. We aimed to investigate the association between BMLs, bone mineral density (BMD), and bone markers in women without radiographic evidence of KOA.

**Methods:**

This single-center cross-sectional study in a Japanese population included 266 women without radiographic evidence of KOA, which was defined as a Kellgren-Lawrence grade < 2. All participants underwent coronal and sagittal T2-weighted fat-suppressed magnetic resonance imaging of their right knee. BML severity was scored according to the Whole-Organ MRI Scoring method. BMD was measured by dual-energy X-ray absorptiometry of the forearm. Levels of bone markers (bone-alkaline phosphatase [BAP], type I procollagen N-terminal propeptide [PINP], cross-linked N-telopeptide of type I collagen [NTx], and tartrate-resistant acid phosphatase-5b [TRACP-5b]), pentosidine, and homocysteine were assessed in the serum. Knee symptoms were evaluated on the basis of the Knee injury and Osteoarthritis and Outcome Score (KOOS). Participants were divided into symptomatic knee and asymptomatic knee groups on the basis of their KOOS according to the classification criteria for early KOA. Multiple linear regression analysis was performed to evaluate the relationship between BMLs, BMD, and bone markers.

**Results:**

The prevalence of BML was 35.3%. Age and some bone marker levels (BAP, PINP, NTx, and TRACP-5b) were higher, and all KOOS subscale scores and BMD were lower in participants with BMLs than in those without BMLs. On multiple linear regression analysis, BMD was negatively associated with BMLs (*p* = 0.014) in participants with symptomatic knees. There was no such association in participants with asymptomatic knees (*p* = 0.918). Among the bone markers, BAP (*p* = 0.006) and PINP (*p* = 0.043) were positively associated with BMLs in participants with symptomatic knees, while BAP (*p* = 0.038) and TRACP-5b (*p* = 0.011) were positively associated with BMLs in participants with asymptomatic knees.

**Conclusions:**

In symptomatic Japanese women without radiographic evidence of KOA, BMD is negatively associated and some bone markers are positively associated with BMLs after adjustment for age and BMI. Thus, maintaining systemic bone metabolism could contribute to BML prevention in patients with pre-radiographic KOA.

## Background

Knee osteoarthritis (KOA) is a major public health problem in the middle-aged to elderly population that negatively affects patients’ activities of daily living (ADL) and quality of life (QOL) [1–3]. Early diagnosis and treatment of KOA are required to prolong lifespan and reduce health care burden. Recently, the concept of early KOA has attracted attention in order to prevent disease development and progression [4, 5]. This concept is characterized by both the presence of knee symptoms and the absence of definitive radiographic abnormalities (Kellgren-Lawrence grades 0–1). Adults with KOA are more likely to report greater knee symptoms the year before they develop radiographic evidence of KOA [6, 7]. On the basis of these evidences, patients with early KOA may need to be targeted for therapeutic interventions as early as possible.

Pathologic changes found in osteoarthritic joints include degradation of the articular cartilage, thickening of the subchondral bone, osteophyte formation, varying degrees of synovial inflammation, degeneration of ligaments and menisci, and hypertrophy of the knee joint capsule [8]. During the disease course, KOA is not only associated with cartilage degradation but also changes in bone tissue. A previous study evaluated subchondral bone marrow lesions (BMLs), detected by magnetic resonance imaging (MRI) among patients with KOA [9]. More recent studies showed that the presence of BMLs is associated with knee pain and predicts cartilage loss in patients with established KOA [10–12]. Upon analysis of histological samples from end-stage KOA patients, BMLs indicated various pathologies, such as bone marrow necrosis, edema, fibrosis, trabecular abnormalities, and bony remodeling [13]. In addition, antiresorptive drugs (e.g., bisphosphonate) were found to reduce the size of BMLs and the risk for total knee arthroplasty in KOA patients [14, 15]. BMLs reflect bony damage, which is associated with knee symptoms in KOA patients and changes its appearance according to bone metabolism such as bone absorption. Despite these pathological relationships among BMLs, bone abnormality, and bone metabolism in established KOA, little is known about how the condition of bone tissue correlates with the appearance of BMLs in early stages.

Bone mineral density (BMD) and bone markers are established tools for evaluating the condition of bone tissues, especially in osteoporosis. However, it remains controversial how BMD affects osteoarthritic changes of the knee joint. In fact, previous studies have reported that both low and high BMDs were associated with the precursors of cartilage degeneration or marginal osteophyte formation [16–18]. Additionally, although many biomechanical markers have been associated with KOA incidence and progression [19, 20], few reports clarify the relationship between bone markers and subchondral bone abnormalities in both established and early KOA patients. Moreover, limited data exist on the direct association among BMLs, BMD, and bone markers, especially in the context of early KOA. Therefore, this study aimed to clarify the cross-sectional association between BMLs, BMD, and serum bone markers in middle-aged women without radiographic features of KOA. Our hypothesis was that lower BMD is associated with symptomatic BMLs, owing to the fragility of subchondral bone and high turnover of bone metabolism.

## Methods

The participants had previously volunteered for the Iwaki Health Promotion Project, which is a community-based preventative medicine program that aims to improve the average life expectancy by conducting general health checkups and prophylactic interventions, as previously described [21–23]. The Ethics Committee of the Hirosaki University Graduate School of Medicine approved the study (reference number: 2017-026), and all subjects gave written informed consent before participation.

A total of 1073 volunteers (441 men and 632 women) enrolled in the project in 2017. The participants answered questionnaires about their past and present medical history, lifestyle, occupation, family history, presence of menopause, health-related QOL, and disease-specific information such as knee symptoms. In this study, we focused on women without radiographic abnormalities because the prevalence of KOA is higher in women [1, 24]. Moreover, 441 men and 144 women who had radiographic features of KOA, 4 women who had undergone knee arthroplasty, 22 participants who did not receive radiography, and 196 women who lacked knee magnetic resonance images; had metal artifacts, a history of rheumatoid arthritis, or meniscus injury; or receiving some types of medications for osteoporosis were excluded. Finally, 266 women without radiographic abnormalities were included in the current analysis.

Knee symptoms were scored using a patient-based outcome score, the Knee injury and Osteoarthritis Outcome Score (KOOS). All participants completed the KOOS questionnaire independently. The KOOS questionnaire is a 42-item, knee-specific, self-administered questionnaire with 5 subscales: pain, symptoms, ADL, sports and recreation (sports), and knee-related QOL. All items were scored from 0 to 4 and then summed. Next, the raw scores were transformed to a 0–100 scale, with 100 representing the best result and 0 representing the worst [25, 26]. Participants were divided into symptomatic knee and asymptomatic knee groups on the basis of their KOOS: a knee was defined as symptomatic when the scores for 2 of the 4 KOOS subscales (except KOOS sports) were “positive” (below 85%), according to the established classification criteria for diagnosing early KOA [5].

The presence of KOA was evaluated by weight-bearing and anterior-posterior radiographs of both knees. The beam was positioned parallel to the floor, with no angle, and aimed at the joint space. The severity of KOA was classified according to the Kellgren-Lawrence (KL) classification [27]. A diagnosis of KOA was defined by KL grade ≥ 2 in the most affected knee. All joints were graded by two orthopedic surgeons (DC and ES), and any discrepancy was resolved by mutual consultation. As aforementioned, only the subjects who had no radiographic evidence of KOA were included in this analysis.

**Fig. 1:**
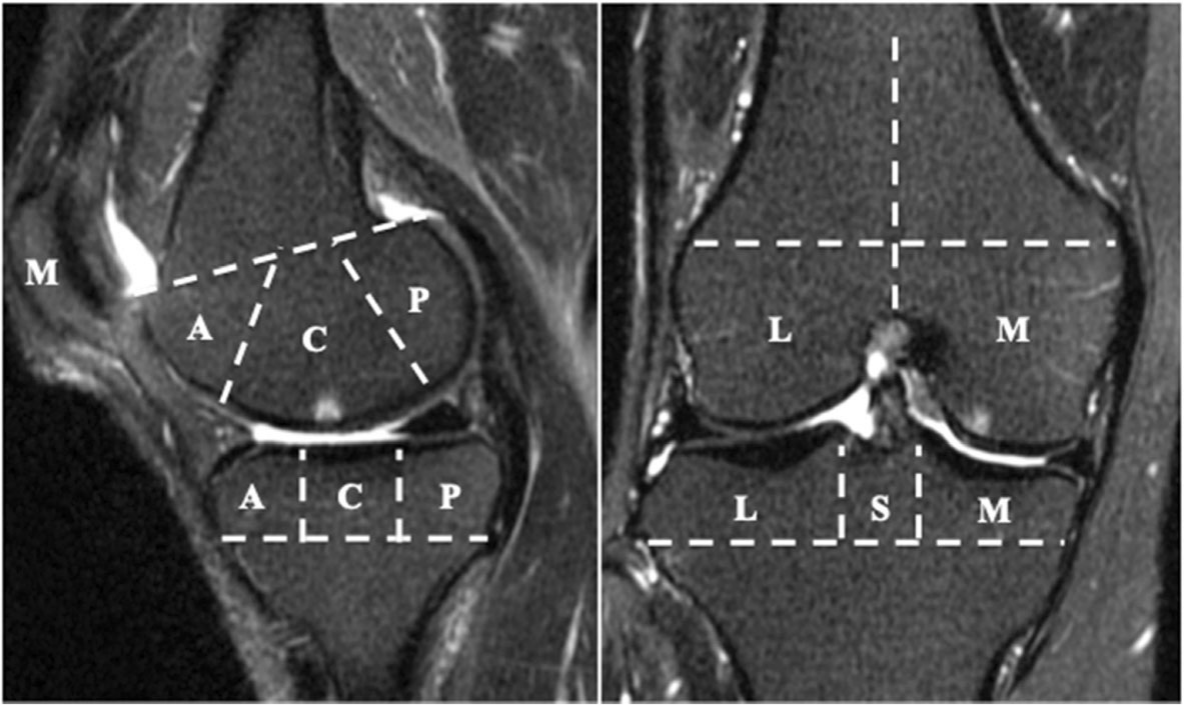
Regional subdivision of the articular surfaces for the assessment of bone marrow lesions (BMLs). BMLs were assessed by the Whole-Organ MRI Scoring (WORMS) method. The patella, femur, and tibia were divided into medial (M) and lateral (L) regions. Region S represents the portion of the tibia beneath the tibial spines. The femoral and tibial surfaces were further subdivided into anterior (A), central (C), and posterior (P) regions

**Fig. 2:**
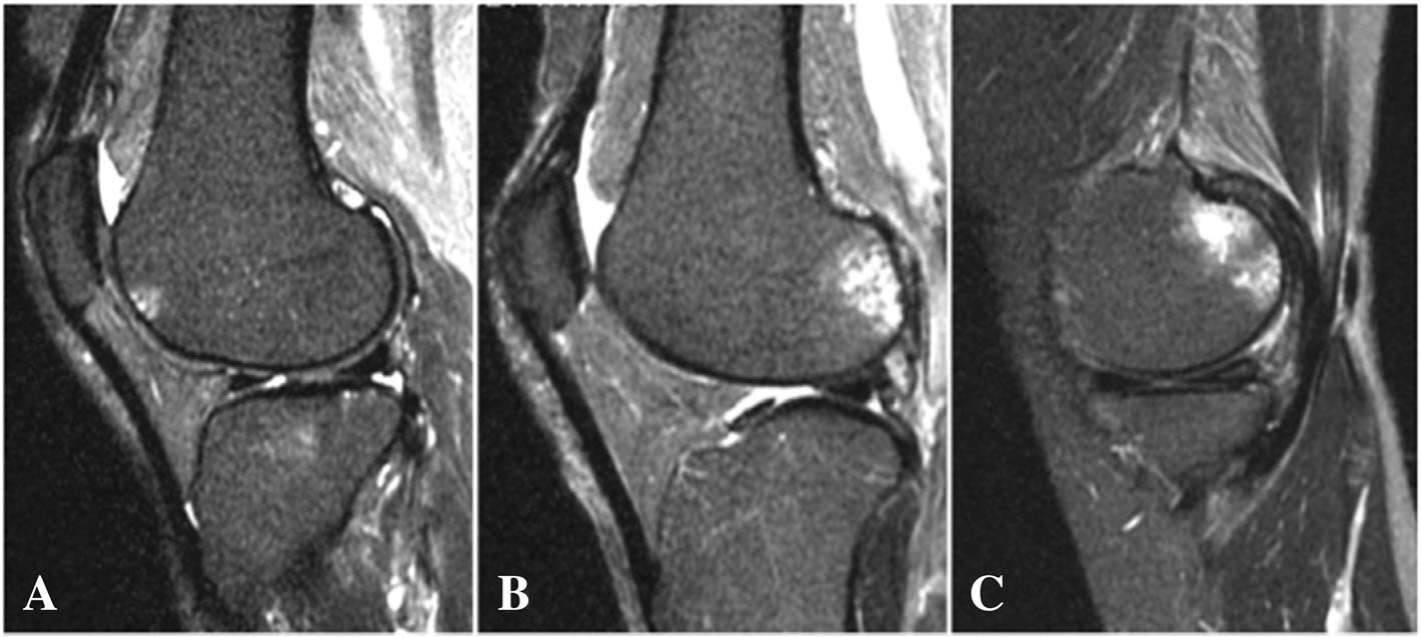
Evaluation of bone marrow lesions (BMLs). BMLs were measured using the coronal and sagittal T2-weighted images and marked by the increased signal intensity area in the subchondral bone. BMLs were graded according to the percentage of the area according to the Whole-Organ MRI Scoring method. **a** Grade 1 BML in the anterior lateral femur. **b** Grade 2 BML in the posterior lateral femur. **c** Grade 3 BML in the posterior medial femur

All participants underwent MRI of the right knee using a rapid extremity coil and mobile magnetic resonance unit (1.5 T; ECHELON RX, Hitachi, Tokyo, Japan) within 1 week after other examinations. The participants were positioned supine with their knees in full extension. Sequences included sagittal and coronal T2-weighted fat saturation fast spin echo (repetition time 5000 ms; echo time 80 ms; field of view 16 cm; 288 × 288 matrix; slice thickness of 3 mm with a between-slices gap of 1.0 mm). BMLs were defined as the area of an irregular hyperintense signal in the subchondral bone. The area was measured semi-quantitatively using the Whole-Organ MRI Scoring (WORMS) method [28] in 15 subregions. Specifically, the medial and lateral compartments of the tibia and femur were divided into 3 subregions (anterior, central, and posterior), and the tibia had 1 additional subregion, representing the area beneath the tibial spine. The patella was divided into medial and lateral subregions (Fig. 1). BMLs were each scored as integers from 0 to 3, where 0 = normal; 1 = mild, < 25% of the region; 2 = moderate, 25–50% of the region; and 3 = severe, > 50% of the region (Fig. 2). The total BML score, which reflected severity, was calculated as the sum of the 15 subregional scores. BML was considered present if the total BML score was greater than zero. Two independent observers (DC and ES) scored MR images in a blinded fashion with no access to the participants’ clinical information. The inter-rater reliability, expressed as interclass correlation coefficients [ICC] (2.1) of scoring BML on 100 randomly selected MR images, was 0.873 (95% confidence interval [CI] 0.815–0.914, *p* < 0.001).

The BMD of the forearm was determined by dual-energy X-ray absorptiometry using DCS-600EXV (Hitachi Aloka Medical, Tokyo, Japan). The region of interest of the BMD was measured on the non-dominant side at one-third of the distal radius, unless there was a history of previous fracture, in which case the dominant side was measured. Because this study was conducted as a part of a community-based general health check project in the limited space of a public hall, it was difficult to investigate the spinal or hip BMD, which needs a large facility to shield radioactive materials. Therefore, only the forearm was eligible for the measurement of systemic BMD.

Blood samples were taken from all participants before breakfast in the early morning for the determination of bone marker levels. Participants had a fasting restriction of 10 h or more before drawing the blood. We commissioned the LSI Medience Corporation to investigate the bone markers. This company has an ISO-15189-accredited laboratory, in which serum assays were performed under strict conditions. The serum levels of bone-alkaline phosphatase (BAP, μg/L, CLEIA; LSI Medience Corp., Tokyo, Japan) and type I procollagen N-terminal propeptides (PINP, μg/L, ECLIA; LSI Medience Corp., Tokyo, Japan) were measured for the assessment of bone formation [29]. The serum cross-linked N-telopeptide of type I collagen (NTx, nM BCE/L, EIA; LSI Medience Corp., Tokyo, Japan) and tartrate-resistant acid phosphatase-5b (TRACP-5b, mU/dL, EIA; LSI Medience Corp., Tokyo, Japan) levels were measured to analyze the degree of bone turnover [30]. In addition, pentosidine (pmol/mL, HPLC; LSI Medience Corp., Tokyo, Japan) and homocysteine (nmol/mL, LC-MS/MS; LSI Medience Corp., Tokyo, Japan) levels were evaluated to determine the degree of bone fragility [31, 32]. These bone markers are routinely used to evaluate the patient’s bone turnover and bone quality and are recommended as the clinical references for diagnosing or treating osteoporosis as per the guidelines of Japanese Society for Bone and Mineral Research [33]. The collected blood was distributed for refrigeration (samples for PINP and homocysteine) and freezing (samples for BAP, NTx, TRACP-5b, and pentosidine) for about 6 h (Hirosaki, Aomori). Next, the samples were sent to the laboratory (Itabashi, Tokyo) over the course of about half a day. During transport, serum samples for BAP, NTx, TRACP-5b, and pentosidine measurement were stored at − 20 °C and samples for PINP and homocysteine evaluation were stored at 7 °C. After arriving at the laboratory, the measurement started immediately, but it lasted for approximately 1 to 5 months; these samples were stored in controlled conditions under the aforementioned temperatures until the process of measurement was completed. Intra- and inter-assay precisions were 0.9–2.6% and 2.3–3.7%, 0.9–1.6% and 1.1–1.7%, 6.0–11.6% and 6.9–11.1%, 1.6–2.9% and 1.8–7.5%, 2.6–3.9% and 9.2–11.1%, and 1.9–3.9% and 2.6–4.5% for BAP, PINP, NTx, TRACP-5b, pentosidine, and homocysteine, respectively.

Data analysis was conducted using SPSS version 22.0 J (SPSS Inc., Chicago, IL, USA) in a cross-sectional manner. Normal distribution of the data was evaluated by QQ-plots and histograms. The mean values of continuous variables (age, body mass index (BMI), BMD, and bone markers) were compared using Student’s *t* test, as these variables were normally distributed. Conversely, the median values of KOOS and BML score were compared using the Mann-Whitney *U* test, as these variables were not normally distributed. The chi-square test was used to compare the prevalence of symptomatic knees. To evaluate the relationship between BMLs and BMD, we performed multiple linear regression analyses with the BML score as an independent variable and age, BMI, and BMD as dependent variables. Moreover, to evaluate the relationship between BMLs and bone markers, we performed multiple linear regression analyses with the BML score as an independent variable and age, BMI, and bone markers as dependent variables. In addition, subgroup analyses were performed to determine whether there was a difference in the relationship between BMLs and BMD, and BMLs and bone markers, depending on the presence or absence of a symptomatic knee. For these regression analyses, natural log-transformed values of BML scores were used, as BML scores were not normally distributed. *p* values less than 0.05 were considered statistically significant.

## Results

**Fig. 3:**
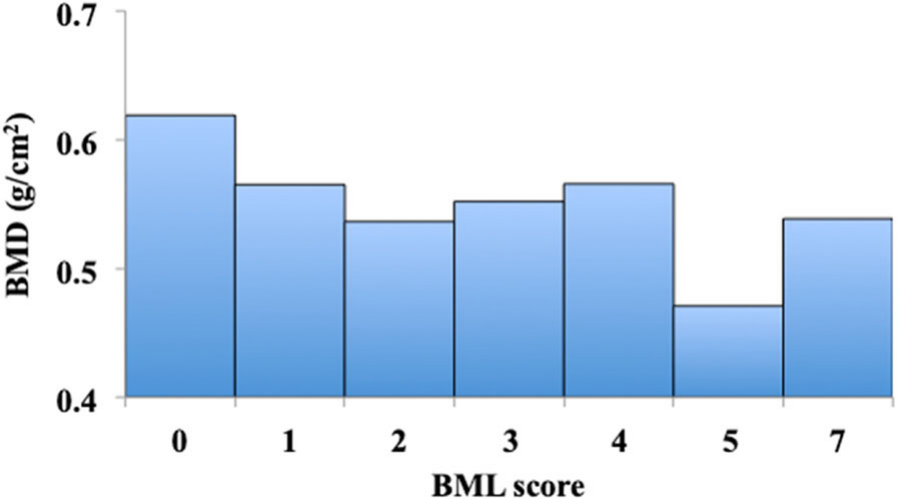
Mean value of BMD for each BML score. BMLs, bone marrow lesions; BMD, bone mineral density

The mean age of study participants was 54.9 ± 9.6 years, mean BMI was 22.2 ± 3.3 kg/m^2^, and mean BMD was 0.60 ± 0.09 g/cm^2^, and 25.6% had symptomatic knee. Ninety-four participants (35.3%) had BMLs in the knee joint. The demographic features of participants with BMLs were higher age, lower KOOS, and lower BMD than those in participants without BMLs. Furthermore, the prevalence of a symptomatic knee among participants with BMLs was significantly higher than that among participants without BMLs (Table 1). The mean BMD tended to decrease as the BML score increased (Fig. 3).

**Table 1:** Clinical characteristics of study participants

	Total sample (*n* = 266)	With BMLs (*n* = 94)	Without BMLs (*n* = 172)	*p* value
Age, years	54.9 ± 9.6	59.8 ± 7.3	52.3 ± 9.7	< 0.001
BMI, kg/m^2^	22.2 ± 3.3	22.3 ± 3.3	22.2 ± 3.3	0.886
KOOS				
Symptom	94.6 (85.7–100.0)	92.9 (82.1–96.4)	96.4 (89.3–100.0)	0.006
Pain	100.0 (88.9–100.0)	95.8 (82.6–100.0)	100.0 (88.9–100.0)	0.013
ADL short ver.	100.0 (92.9–100.0)	100.0 (85.7–100.0)	100.0 (96.4–100.0)	0.037
QOL	87.5 (73.4–100.0)	81.3 (62.5–95.3)	93.8 (75.0–100.0)	0.004
Symptomatic knee, *n* (%)	68 (25.6)	34 (36.2)	34 (19.8)	0.003
BMD, g/cm^2^	0.60 ± 0.09	0.55 ± 0.08	0.62 ± 0.09	< 0.001
BML score	0.0 (0.0–1.0)	1.0 (1.0–2.0)	0.0 (0.0–0.0)	< 0.001

The values represent demographic data of all participants, participants with BMLs, and those without BMLs. Data are presented as mean ± SD for age, BMI, and BMD and median (range) for KOOS and BML score. The data on symptomatic knees is based on the number of participants (percentage of the whole population). Student’s *t* test was used to compare the mean values of age, BMI, and BMD; Mann-Whitney *U* test was used to compare the median values of KOOS and BML score, and chi-square test was used to compare the proportions of symptomatic knees between participants with and without BMLs. *p* value indicates the significance of the difference between participants with and without BMLs

*BMI* body mass index, *KOOS* Knee injury and Osteoarthritis Outcome Score, *BMD* bone mineral density, *BMLs* bone marrow lesions

According to the linear regression analysis, BMD was negatively associated with BML in all participants. However, the adjusted regression model showed that BMD was negatively associated with BML only in participants with a symptomatic knee after adjustment for age and BMI, and this association was attenuated and not statistically significant in participants with an asymptomatic knee (Table 2).

**Table 2:** Relationship between BMLs and forearm BMD

	*R*^2^ (adjusted)	*β*	*p* value	95% CI
Total sample (*n* = 266)	0.110	− 0.100	0.252	− 0.517 to 0.137
With asymptomatic knee (*n* = 198)	0.071	− 0.011	0.918	− 0.399 to 0.360
With symptomatic knee (*n* = 68)	0.222	− 0.355	0.014	− 1.443 to − 0.167

Statistical analysis—multiple linear regression analysis. Dependent variables—the natural logs of BML score plus 5. Independent variables—age, BMI, and BMD

*BMLs* bone marrow lesions, *BMD* bone mineral density, *R*^*2*^ coefficient of determination, *β* standardized regression coefficient, *CI* confidence interval

Serum concentrations of BAP, PINP, NTx, TRACP-5b, and pentosidine were significantly higher in participants with BMLs than in those without BMLs. However, there was no significant difference in homocysteine levels (Table 3). According to the linear regression analysis, BAP, PINP, NTx, and TRACP-5b were positively associated with BML in all participants. Although the adjusted regression model showed that these associations were attenuated, they were still statistically significant (Table 4). In participants with asymptomatic knee, the adjusted regression model showed that BAP and TRACP-5b were significantly associated with BML (Table 5). In participants with symptomatic knee, the adjusted regression model showed that BAP and PINP were significantly associated with BML, while the association between NTx and TRACP-5b was close to being significant (Table 6).

**Table 3:** Laboratory characteristics of study participants

	Total sample	With BMLs (*n* = 94)	Without BMLs (*n* = 172)	*p* value
BAP	13.7 ± 5.0	15.6 ± 5.0	12.6 ± 4.6	< 0.001
PINP	49.2 ± 18.0	55.6 ± 18.9	45.8 ± 16.6	< 0.001
NTx	15.5 ± 5.0	16.7 ± 6.3	14.8 ± 4.1	0.002
TRACP-5b	442.2 ± 195.0	523.2 ± 188.6	397.8 ± 184.4	< 0.001
Pentosidine	29.2 ± 12.4	31.4 ± 17.3	28.1 ± 8.4	0.038
Homocysteine	8.5 ± 2.6	8.7 ± 2.5	8.4 ± 2.6	0.327

Student’s *t* test was used to compare the mean values of BAP, PINP, NTx, TRACP-5b, pentosidine, and homocysteine. *p* value indicates the significance of difference between participants with and without BMLs

*BAP* bone-specific alkaline phosphatase, *BML* bone marrow lesion, *NTx* crosslinked N-telopeptide of type I collagen, *PINP* procollagen type I N-terminal propeptide, *TRACP-5b* tartrate-resistant acid phosphatase-5b, *BML* bone marrow lesion

**Table 4:** Relationships between BMLs and bone markers

Total sample (*n* = 266)	*R*^2^ (adjusted)	*β*	*p* value	95% CI
BAP	0.135	0.193	0.003	0.002 to 0.011
PINP	0.123	0.142	0.022	0.000 to 0.003
NTx	0.121	0.128	0.032	0.000 to 0.009
TRACP-5b	0.131	0.194	0.006	0.000 to 0.000
Pentosidine	0.106	0.011	0.852	− 0.002 to 0.002
Homocysteine	0.106	− 0.022	0.708	− 0.009 to 0.006

Statistical analysis—multiple linear regression analysis. Dependent variables—the natural logs of BML score plus 5. Independent variables—age, BMI, and bone markers (BAP, PINP, NTx, TRACP-5b, pentosidine, and homocysteine)

*BMLs* bone marrow lesions, *BAP* bone-specific alkaline phosphatase, *PINP* procollagen type I N-terminal propeptide, *NTx* crosslinked N-telopeptide of type I collagen, *TRACP-5b* tartrate-resistant acid phosphatase-5b, *R*^*2*^ coefficient of determination, *β* standardized regression coefficient, *CI* confidence interval

**Table 5:** Relationships between BMLs and bone markers in participants with asymptomatic knee

With asymptomatic knee (*n* = 198)	*R*^2^ (adjusted)	*β*	*p* value	95% CI
BAP	0.091	0.167	0.038	0.000 to 0.010
PINP	0.085	0.131	0.087	0.000 to 0.003
NTx	0.075	0.069	0.340	− 0.003 to 0.008
TRACP-5b	0.102	0.228	0.011	0.000 to 0.000
Pentosidine	0.072	0.036	0.623	− 0.001 to 0.002
Homocysteine	0.071	− 0.023	0.742	− 0.010 to 0.007

Statistical analysis—multiple linear regression analysis. Dependent variables—the natural logs of BML score plus 5. Independent variables—age, BMI, and bone markers (BAP, PINP, NTx, TRACP-5b, pentosidine, and homocysteine)

*BMLs* bone marrow lesions, *BAP* bone-specific alkaline phosphatase, *PINP* procollagen type I N-terminal propeptide, *NTx* crosslinked N-telopeptide of type I collagen, *TRACP-5b* tartrate-resistant acid phosphatase-5b, *R*^*2*^ coefficient of determination, *β* standardized regression coefficient, *CI* confidence interval

**Table 6:** Relationships between BMLs and bone markers in participants with symptomatic knee

With symptomatic knee (*n* = 68)	*R*^2^ (adjusted)	*β*	*p* value	95% CI
BAP	0.239	0.314	0.006	0.004 to 0.024
PINP	0.198	0.231	0.043	0.000 to 0.005
NTx	0.189	0.210	0.065	0.000 to 0.013
TRACP-5b	0.188	0.225	0.068	0.000 to 0.000
Pentosidine	0.151	− 0.084	0.482	− 0.008 to 0.004
Homocysteine	0.152	− 0.093	0.440	− 0.025 to 0.011

Statistical analysis—multiple linear regression analysis. Dependent variables—the natural logs of BML score plus 5. Independent variables—age, BMI, and bone markers (BAP, PINP, NTx, TRACP-5b, pentosidine, and homocysteine)

*BMLs* bone marrow lesions, *BAP* bone-specific alkaline phosphatase, *PINP* procollagen type I N-terminal propeptide, *NTx* crosslinked N-telopeptide of type I collagen, *TRACP-5b* tartrate-resistant acid phosphatase-5b, *R*^*2*^ coefficient of determination, *β* standardized regression coefficient, *CI* confidence interval

## Discussion

This study evaluated the association between BMLs, BMD, and bone markers in middle-aged Japanese women with no radiographic evidence of KOA. We found that the prevalence of BML was 35%, and BML in the symptomatic knee was associated with a lower BMD, after adjusting for age and BMI. Furthermore, BAP, PINP, NTx, and TRACP-5b were positively associated with the presence of BML in all participants, while only BAP and PINP were positively associated with BML in participants with symptomatic knees.

Few studies have investigated the prevalence of BMLs specifically in a population without radiographic abnormalities. Laberge et al. reported that the prevalence of BML was 46.7% in a middle-aged population with knee symptoms but no radiographic evidence of KOA [34]. Sowers et al. found that BMLs were identified in 40% knees of subjects without radiographic evidence of KOA [35]. In this study, the prevalence of BML was 35% and slightly lower than that in previous reports. This may be because participants in this study had fewer knee symptoms compared to the participants of past reports.

The pathological association between KOA and BMD has remained controversial. Many previous studies have reported an association between high femoral BMD (weight-bearing) and KOA [16, 36]. However, another study indicated that high BMD decreased the risk of progression of radiographic KOA, but it may be associated with an increased risk of incident KOA [37]. Moreover, one study suggested that the relationship between BMD and KOA varies depending on the site of OA and measurement of BMD [18]. These results suggested that the relationship between BMD and KOA might differ depending on the disease stage of KOA (from early stage to end stage) and the presence or absence of loading on the measurement site of BMD. In this study, low BMD in the forearm (non-weight-bearing) and high bone marker levels were associated with BMLs. This finding suggests that systemic bone fragility may result in early subchondral changes such as microcracking before radiologic osteoarthritic findings become definitive. Laslett et al. reported that the size of BML shrinks due to the bisphosphonates used for osteoporosis treatment, resulting in improved pain [14]. In addition, Zanetti et al. reported that the pathology of BML includes necrotic or remodeled trabeculae [13]. Therefore, we believe in a possibility that bisphosphonate administration may be useful for BML before radiographic abnormalities become apparent.

There are many reports on the relationships between KOA and bone markers. Kumm et al. showed that PINP had a diagnostic and predictive value for knee OA progression [38]. Nwosu et al. reported that serum TRACP-5b activity was associated with baseline pain and changes in pain over 3 years [39]. Kraus et al. found that serum NTx levels were associated with subject cases that had both progressive pain and radiographic progression of knee OA over a 4-year period [40]. On the other hand, only a few studies in addition to this study have investigated the association between BMLs and bone markers. Hunter et al. [41] showed that serum NTx was weakly associated with the presence of BMLs, whereas Deveza et al. [42] reported that serum CTX-I and urinary CTX-Ia levels were significantly associated with large BMLs. These results suggest an acceleration of bone turnover in KOA patients with BMLs. In this study, bone markers (BAP, PINP, NTx, and TRACP-5b) were positively associated with BMLs. Our results indicate that BMLs reflect enhanced bone remodeling to repair microfractures in the early stages of KOA. In particular, the correlation coefficient of BAP to BML was higher than that of other bone markers. There are a few studies reporting the relationship between BAP and KOA. BAP is one of the bone formation markers and reflects the activity of osteoblasts. A previous study found that the subchondral bone in osteoarthritic knee joints changed its phenotypic expression of osteoblasts [43, 44]. On the basis of the current results, the elevation in serum BAP concentration reflects that BMLs have a potential to activate osteoblasts in the subchondral bone of knee joints in the early stages of KOA.

Interestingly, in participants with an asymptomatic knee, there was no association between BMLs and BMD after adjustment for age and BMI, in contrast to that in participants with a symptomatic knee. A few reports have shown that early changes in the subchondral bone such as microcracking and periarticular osteoporosis occur before cartilage loss [45, 46]. Furthermore, several observational studies have explored knee pain and other symptoms as predictors of future radiographic KOA [6, 47, 48]. In this study, because women with a symptomatic knee might be at a higher risk of cartilage loss and incidence of radiographic KOA, they showed a stronger relationship between BMD and BMLs than did women with an asymptomatic knee. These results indicate that future studies will need to determine the importance of maintaining systemic bone metabolism in women without radiographic abnormalities who have knee symptoms.

This study has several limitations. First, we did not evaluate BMD of the femoral neck and lumbar spine, which are considered the gold standards for diagnosing osteoporosis to determine fracture risk. This standard method needs cost, space, and frequent calibration to maintain its property. Because of these reasons, we could not evaluate the BMD of weight-bearing sites during the current study. Evaluation of forearm BMD determined the bone status of non-weight-bearing sites; this discrepancy could affect the current results. Second, all participants in this study were women. Although the prevalence of KOA is higher in women than in men, men may be more likely to experience a knee trauma, which may affect the prevalence of BMLs. Third, we did not evaluate knee alignment, which might affect the association of abnormal loading in the knee with the presence of BMLs. Fourth, because of the exploratory nature of this study, adjustments for multiple testing were not carried out. Therefore, these findings should be viewed as trends that need to be further investigated. Despite these limitations, this study shows the relationships between BML, BMD, and bone markers. In particular, BMD was negatively associated with BMLs, after adjustments for age and BMI, only in participants with symptomatic knees. The findings of this study were based on cross-sectional data; therefore, future longitudinal studies are warranted to investigate the causal relationship between BMD and BMLs. Furthermore, it is necessary to investigate whether BMLs resolve after treatment with antiresorptive drugs and whether the incidence and progression of KOA can be prevented in large observational studies.

## Conclusion

A lower BMD was associated with the presence of BMLs in symptomatic knees, even if the knee was radiographically normal. In these knees, a high turnover of bone metabolism was related to the presence of BMLs.

## Abbreviations

ADL: Activities of daily living
BAP: Bone-alkaline phosphatase
BMI: Body mass index
BML: Bone marrow lesions
CI: Confidence interval
ICC: Interclass correlation coefficients
KL: Kellgren-Lawrence classification
KOA: Knee osteoarthritis
KOOS: Knee injury and Osteoarthritis Outcome Score
NTx: Cross-linked N-telopeptide of type I collagen
PINP: Type I procollagen N-terminal propeptides
QOL: Quality of life
TRACP-5b: Tartrate-resistant acid phosphatase-5b
WORMS: Whole-Organ MRI Scoring

## References

[1] Yoshimura N, Muraki S, Oka H, Mabuchi A, En-Yo Y, Yoshida M (2009). Prevalence of knee osteoarthritis, lumbar spondylosis, and osteoporosis in Japanese men and women: the research on osteoarthritis/osteoporosis against disability study. J Bone Miner Metab.

[2] Cross M, Smith E, Hoy D, Nolte S, Ackerman I, Fransen M (2014). The global burden of hip and knee osteoarthritis: estimates from the global burden of disease 2010 study. Ann Rheum Dis.

[3] Kluzek S, Sanchez-Santos MT, Leyland KM, Judge A, Spector TD, Hart D (2016). Painful knee but not hand osteoarthritis is an independent predictor of mortality over 23 years follow-up of a population-based cohort of middle-aged women. Ann Rheum Dis.

[4] Madry H, Kon E, Condello V, Peretti GM, Steinwachs M, Seil R (2016). Early osteoarthritis of the knee. Knee Surg Sports Traumatol Arthrosc.

[5] Luyten FP, Bierma-Zeinstra S, Dell’Accio F, Kraus VB, Nakata K, Sekiya I (2018). Toward classification criteria for early osteoarthritis of the knee. Semin Arthritis Rheum.

[6] Case R, Thomas E, Clarke E, Peat G (2015). Prodromal symptoms in knee osteoarthritis: a nested case-control study using data from the Osteoarthritis Initiative. Osteoarthr Cartil.

[7] Driban JB, Price LL, Eaton CB, Lu B, Lo GH, Lapane KL (2016). Individuals with incident accelerated knee osteoarthritis have greater pain than those with common knee osteoarthritis progression: data from the Osteoarthritis Initiative. Clin Rheumatol.

[8] Loeser RF, Goldring SR, Scanzello CR, Goldring MB (2012). Osteoarthritis: a disease of the joint as an organ. Arthritis Rheumatol.

[9] Felson DT, Chaisson CE, Hill CL, Totterman SM, Gale ME, Skinner KM (2001). The association of bone marrow lesions with pain in knee osteoarthritis. Ann Intern Med.

[10] Kothari A, Guermazi A, Chmiel JS, Dunlop D, Song J, Almagor O (2010). Within-subregion relationship between bone marrow lesions and subsequent cartilage loss in knee osteoarthritis. Arthritis Care Res (Hoboken)..

[11] Driban JB, Lo GH, Lee JY, Ward RJ, Miller E, Pang J (2011). Quantitative bone marrow lesion size in osteoarthritic knees correlates with cartilage damage and predicts longitudinal cartilage loss. BMC Musculoskelet Disord.

[12] Driban JB, Price L, Lo GH, Pang J, Miller E, Ward R (2013). Evaluation of bone marrow lesion volume as a knee osteoarthritis biomarker--longitudinal relationships with pain and structural changes: data from the Osteoarthritis Initiative. Arthritis Res Ther.

[13] Zanetti M, Bruder E, Romero J, Hodler J (2000). Bone marrow edema pattern in osteoarthritic knees: correlation between MR imaging and histologic findings. Radiology..

[14] Laslett LL, Doré DA, Quinn SJ, Boon P, Ryan E, Winzenberg TM (2012). Zoledronic acid reduces knee pain and bone marrow lesions over 1 year: a randomised controlled trial. Ann Rheum Dis.

[15] Fu SH, Wang CY, Yang RS, Wu FL, Hsiao FY (2017). Bisphosphonate use and the risk of undergoing total knee arthroplasty in osteoporotic patients with osteoarthritis: a nationwide cohort study in Taiwan. J Bone Joint Surg Am.

[16] Hannan MT, Anderson JJ, Zhang Y, Levy D, Felson DT (1993). Bone mineral density and knee osteoarthritis in elderly men and women. The Framingham Study. Arthritis Rheumatol..

[17] Hart DJ, Mootoosamy I, Doyle DV, Spector TD (1994). The relationship between osteoarthritis and osteoporosis in the general population: the Chingford Study. Ann Rheum Dis.

[18] Hochberg MC, Lethbridge-Cejku M, Tobin JD (2004). Bone mineral density and osteoarthritis: data from the Baltimore Longitudinal Study of Aging. Osteoarthr Cartil.

[19] van Spil WE, DeGroot J, Lems WF, Oostveen JC, Lafeber FP (2010). Serum and urinary biochemical markers for knee and hip-osteoarthritis: a systematic review applying the consensus BIPED criteria. Osteoarthr Cartil.

[20] Lafeber FP, van Spil WE (2013). Osteoarthritis year 2013 in review: biomarkers; reflecting before moving forward, one step at a time. Osteoarthr Cartil.

[21] Inoue R, Ishibashi Y, Tsuda E, Yamamoto Y, Matsuzaka M, Takahashi I (2011). Knee osteoarthritis, knee joint pain and aging in relation to increasing serum hyaluronan level in the Japanese population. Osteoarthr Cartil.

[22] Sasaki E, Tsuda E, Yamamoto Y, Maeda S, Inoue R, Chiba D (2015). Serum hyaluronic acid concentration predicts the progression of joint space narrowing in normal knees and established knee osteoarthritis - a five-year prospective cohort study. Arthritis Res Ther..

[23] Chiba D, Maeda S, Sasaki E, Ota S, Nakaji S, Tsuda E (2017). Meniscal extrusion seen on ultrasonography affects the development of radiographic knee osteoarthritis: a 3-year prospective cohort study. Clin Rheumatol.

[24] Felson DT, Naimark A, Anderson J, Kazis L, Castelli W, Meenan RF (1987). The prevalence of knee osteoarthritis in the elderly. The Framingham Osteoarthritis Study. Arthritis Rheum.

[25] Roos EM, Roos HP, Lohmander LS, Ekdahl C, Beynnon BD (1998). Knee Injury and Osteoarthritis Outcome Score (KOOS)--development of a self-administered outcome measure. J Orthop Sports Phys Ther.

[26] Nakamura N, Takeuchi R, Sawaguchi T, Ishikawa H, Saito T, Goldhahn S (2011). Cross-cultural adaptation and validation of the Japanese Knee Injury and Osteoarthritis Outcome Score (KOOS). J Orthop Sci.

[27] Kellgren JH, Lawrence JS (1957). Radiological assessment of osteo-arthrosis. Ann Rheum Dis.

[28] Peterfy CG, Guermazi A, Zaim S, Tirman PF, Miaux Y, White D (2004). Whole-Organ Magnetic Resonance Imaging Score (WORMS) of the knee in osteoarthritis. Osteoarthr Cartil.

[29] Delmas PD, Eastell R, Garnero P, Seibel MJ, Stepan J (2000). The use of biochemical markers of bone turnover in osteoporosis. Committee of Scientific Advisors of the International Osteoporosis Foundation. Osteoporos Int.

[30] Garnero P (2001). Markers of bone turnover in prostate cancer. Cancer Treat Rev.

[31] Saito M, Marumo K (2010). Collagen cross-links as a determinant of bone quality: a possible explanation for bone fragility in aging, osteoporosis, and diabetes mellitus. Osteoporos Int.

[32] Shiraki M, Kuroda T, Shiraki Y, Tanaka S, Higuchi T, Saito M (2011). Urinary pentosidine and plasma homocysteine levels at baseline predict future fractures in osteoporosis patients under bisphosphonate treatment. J Bone Miner Metab.

[33] Nishizawa Y, Ohta H, Miura M, Inaba M, Ichimura S, Shiraki M (2013). Guidelines for the use of bone metabolic markers in the diagnosis and treatment of osteoporosis (2012 edition). J Bone Miner Metab.

[34] Laberge MA, Baum T, Virayavanich W, Nardo L, Nevitt MC, Lynch J (2012). Obesity increases the prevalence and severity of focal knee abnormalities diagnosed using 3T MRI in middle-aged subjects--data from the Osteoarthritis Initiative. Skelet Radiol.

[35] Sowers MF, Hayes C, Jamadar D, Capul D, Lachance L, Jannausch M (2003). Magnetic resonance-detected subchondral bone marrow and cartilage defect characteristics associated with pain and X-ray-defined knee osteoarthritis. Osteoarthr Cartil.

[36] Burger H, van Daele PL, Odding E, Valkenburg HA, Hofman A, Grobbee DE (1996). Association of radiographically evident osteoarthritis with higher bone mineral density and increased bone loss with age. The Rotterdam Study. Arthritis Rheum.

[37] Zhang Y, Nevitt M, Niu J, Lewis C, Torner J, Guermazi A (2011). Fluctuation of knee pain and changes in bone marrow lesions, effusions, and synovitis on magnetic resonance imaging. Arthritis Rheum.

[38] Kumm J, Tamm A, Lintrop M, Tamm A (2013). Diagnostic and prognostic value of bone biomarkers in progressive knee osteoarthritis: a 6-year follow-up study in middle-aged subjects. Osteoarthr Cartil.

[39] Nwosu LN, Allen M, Wyatt L, Huebner JL, Chapman V, Walsh DA (2017). Pain prediction by serum biomarkers of bone turnover in people with knee osteoarthritis: an observational study of TRAcP5b and cathepsin K in OA. Osteoarthr Cartil.

[40] Kraus VB, Collins JE, Hargrove D, Losina E, Nevitt M, Katz JN (2017). Predictive validity of biochemical biomarkers in knee osteoarthritis: data from the FNIH OA Biomarkers Consortium. Ann Rheum Dis.

[41] Hunter DJ, Lavalley M, Li J, Baur D, Nevitt M, Groot D (2008). Biochemical markers of bone turnover and their association with bone marrow lesions. Arthritis Res Ther.

[42] Deveza LA, Kraus VB, Collins JE, Guermazi A, Roemer FW, Bowes M (2017). Association between biochemical markers of bone turnover and bone changes on imaging: data from the osteoarthritis initiative. Arthritis Care Res (Hoboken).

[43] Hilal G, Martel-Pelletier J, Pelletier JP, Ranger P, Lajeunesse D (1998). Osteoblast-like cells from human subchondral osteoarthritic bone demonstrate an altered phenotype in vitro: possible role in subchondral bone sclerosis. Arthritis Rheum.

[44] Bailey AJ, Sims TJ, Knott L (2002). Phenotypic expression of osteoblast collagen in osteoarthritic bone: production of type I homotrimer. Int J Biochem Cell Biol.

[45] Karvonen RL, Miller PR, Nelson DA, Granda JL, Fernández-Madrid F (1998). Periarticular osteoporosis in osteoarthritis of the knee. J Rheumatol.

[46] Buckland-Wright C (2004). Subchondral bone changes in hand and knee osteoarthritis detected by radiography. Osteoarthr Cartil.

[47] Kastelein M, Luijsterburg PA, Belo JN, Verhaar JA, Koes BW, Bierma-Zeinstra SM (2011). Six-year course and prognosis of nontraumatic knee symptoms in adults in general practice: a prospective cohort study. Arthritis Care Res (Hoboken)..

[48] Hensor EM, Dube B, Kingsbury SR, Tennant A, Conaghan PG (2015). Toward a clinical definition of early osteoarthritis: onset of patient-reported knee pain begins on stairs. Data from the osteoarthritis initiative. Arthritis Care Res (Hoboken)..

